# Faculty Development for High‐Acuity Procedures: A Train‐the‐Trainer Simulation‐Based Innovation for Transvenous Pacemaker Placement

**DOI:** 10.1002/aet2.70236

**Published:** 2026-09-01

**Authors:** Jessica Baez, Erin McDonough, Heidi Sucharew, Sally A. Santen, Elizabeth Leenellett

**Affiliations:** ^1^ Department of Emergency Medicine University of Cincinnati Cincinnati Ohio USA

## Abstract

**Background:**

Emergency medicine faculty are expected to perform and supervise high‐acuity, low‐occurrence (HALO) procedures despite limited clinical opportunities, placing them at risk for procedural skill decay. Transvenous pacemaker (TVP) placement is a life‐saving but infrequently performed intervention. While simulation is widely used for trainees, sustainable faculty development models for procedural maintenance remain limited.

**Methods:**

We developed a train‐the‐trainer, peer‐led, simulation‐based faculty development program focused on TVP placement within a single academic emergency department. Volunteer faculty trainers received focused preparation in TVP placement, simulator use, and facilitation strategies, and subsequently led small‐group refresher sessions using a low‐cost, reusable TVP simulator. The intervention was embedded within existing departmental educational venues and incentivized through continuing medical education credit and value‐based compensation alignment. Over 1 year, 12 sessions (30–60 min each) were conducted, capped at four learners per session and facilitated by 10 faculty trainers. Faculty completed pre‐ and post‐session surveys assessing prior experience and self‐reported comfort with TVP placement. Paired pre‐ and post‐intervention comfort scores were compared.

**Results:**

Fifty‐four of 56 eligible faculty participated and completed paired surveys. Baseline exposure to TVP placement was limited, with 37% reporting no prior simulation experience and 24% reporting no prior live placement. Faculty reporting comfort or high comfort with independently placing a TVP increased from 14.8% pre‐intervention to 83.3% post‐intervention. Mean comfort scores improved from 2.1 ± 0.6 to 3.2 ± 0.6 on a 4‐point Likert scale (*p* < 0.001). The sessions were rated as highly useful (mean 4.9 ± 0.3 on a 5‐point Likert scale), and all participants would recommend the training to colleagues.

**Conclusion:**

A train‐the‐trainer, peer‐led simulation model significantly improved faculty comfort with transvenous pacemaker placement while minimizing reliance on centralized simulation resources. Embedding brief, small‐group sessions within existing educational structures offers a feasible and scalable approach for maintaining faculty competency in HALO procedures.

## 1 Need for Innovation

Emergency medicine faculty are expected to competently perform and supervise high‐acuity, low‐occurrence (HALO) procedures, despite limited opportunities for hands‐on clinical practice. Transvenous pacemaker (TVP) placement exemplifies this challenge because it is a life‐saving intervention for unstable bradyarrhythmias and complete heart block. However, it is infrequently performed by most academic emergency physicians [1, 2, 3, 4]. The resulting risk of procedural skill decay has been well described and represents both a professional responsibility concern and a patient safety issue [5, 6, 7, 8, 9].

Needs assessments of academic emergency medicine faculty demonstrate inconsistent approaches to maintaining procedural skills and highlight limited formal structures for procedural competency maintenance [10]. Although simulation is widely used in resident education, far fewer structured mechanisms exist to support ongoing procedural competency among faculty [9]. Traditional faculty procedural refreshers often rely on centralized expertise, dedicated simulation staff, or external courses, limiting scalability and sustainability [11]. These models can be costly, time‐intensive, and difficult to integrate into busy academic environments. As a result, faculty development in procedural skills is often ad hoc, optional, or absent altogether.

This gap is particularly concerning given national calls emphasizing physicians' responsibility to maintain competence throughout their careers [6, 7]. There is a need for innovative faculty development models that are scalable, resource‐efficient, and embedded within existing departmental structures. A train‐the‐trainer, peer‐led simulation model represents a promising but underexplored approach to addressing this need in academic emergency medicine.

## 2 Background

Simulation‐based interventions have been proposed as one strategy to mitigate procedural skill decay for infrequently performed procedures [7, 8]. Academic emergency medicine faculty may be uniquely vulnerable due to increasing supervisory roles and declining direct procedural exposure over time [9].

Simulation‐based education provides a controlled environment for deliberate practice, repetition, and feedback, which are key components associated with procedural skill acquisition and retention [12, 13]. Just‐in‐time simulation interventions have also been shown to improve procedural performance despite skill decay, supporting simulation as an effective strategy for maintaining competence in infrequently performed procedures [14]. Prior work has demonstrated that simulation‐based mastery learning improves emergency medicine residents' ability to perform TVP placement [15, 16]. However, most simulation curricula focus on trainees, and less attention has been paid to how faculty themselves can be supported as learners, particularly in ways that are feasible across an entire department.

Faculty development literature highlights several barriers to participation, including time constraints, lack of perceived relevance, and limited access to educational resources [17]. Peer‐led educational models may help overcome these barriers by promoting autonomy, relevance, and social relatedness [18]. Self‐determination theory suggests that learning environments supporting these elements enhance motivation and engagement, making peer‐to‐peer instruction particularly well suited for faculty development [18].

Train‐the‐trainer models have been used in other educational contexts to disseminate expertise efficiently, yet they remain underutilized in procedural faculty development. Applying this model to simulation‐based HALO procedure training offers an opportunity to decentralize expertise, normalize deliberate practice among faculty, and create sustainable systems for maintaining procedural readiness.

## 3 Objective of the Innovation

The objective of this innovation was to develop, implement, and evaluate a train‐the‐trainer, peer‐led, simulation‐based faculty development model designed to mitigate procedural skill decay and improve faculty comfort with transvenous pacemaker placement. TVP placement was selected as the target procedure because it is a technically complex HALO procedure that emergency physicians are expected to perform despite infrequent clinical opportunities, making it an ideal proof‐of‐concept application for this train‐the‐trainer model. Specifically, we sought to determine whether a peer‐led refresher program, delivered by faculty trainers using a low‐cost simulator and embedded within existing departmental educational forums, would improve self‐reported faculty comfort with independently performing TVP placement.

## 4 Development Process

We designed a structured faculty development initiative centered on a train‐the‐trainer instructional framework. Rather than relying on a small group of simulation experts or educational leaders, an open call for volunteer faculty trainers was distributed to the department. Any faculty member was eligible to volunteer, with the goals of distributing educational responsibilities beyond traditional education faculty, increasing departmental ownership of procedural competency, and creating opportunities for educational leadership. Ten faculty members ultimately volunteered to serve as trainers.

Trainer preparation was conducted by two faculty champions (JB and EM) through individualized train‐the‐trainer sessions conducted over time prior to departmental implementation. Each session lasted approximately 30 min and included review of transvenous pacemaker placement, hands‐on practice with the simulation model, simulator assembly and troubleshooting, and discussion of facilitation strategies for leading small‐group refresher sessions. Trainers were also introduced to standardized teaching pearls, including practical approaches to pacing wire orientation, use of the pacemaker generator, and troubleshooting techniques intended to simplify the procedure and improve learner confidence.

The intervention utilized a reusable, cost‐effective TVP simulation model adapted from a conceptual design previously described [19]. The model was selected for its balance of conceptual accuracy, affordability, and durability, allowing repeated use without significant ongoing costs.

To promote consistency across trainers, all trainers received a standardized written trainer guide that included step‐by‐step instructions for simulator assembly, equipment setup, procedural performance, and session facilitation. A dedicated mobile simulation cart containing all required equipment was assembled to allow trainers to transport the simulator to educational venues and prepare the station within minutes. To maximize clinical fidelity, training utilized the same transvenous pacemaker generator used in routine emergency department practice. During implementation, use of the clinical equipment identified inconsistencies in available transvenous pacing supplies across clinical sites, prompting standardization of equipment used in patient care. Design considerations emphasized feasibility, reproducibility, and scalability, ensuring that the model could be easily adopted by other institutions. Standardized trainer materials allowed multiple faculty trainers to independently facilitate sessions while maintaining consistency in instructional content and learner experience. The trainer guide is available as Data S1 to facilitate replication by other institutions.

Faculty training sessions were intentionally concise and emphasized operation of the pacing generator, transvenous pacing wire insertion (floating the pacer) and positioning, equipment setup, troubleshooting, and common procedural pitfalls. Although faculty could practice central line introducer placement if desired, the primary educational emphasis was on the components of TVP placement most likely to challenge practicing emergency physicians.

Faculty trainers led small‐group sessions emphasizing deliberate practice, stepwise instruction, and real time feedback. This peer‐led approach was designed to reduce hierarchy, foster psychological safety, and encourage open discussion of uncertainty, an important consideration for attending physicians engaging in skill refreshers.

## 5 Implementation Phase

The innovation was implemented within a single academic emergency medicine department and embedded into existing educational venues, including faculty meetings, retreats, and grand rounds. Training sessions were intentionally scheduled immediately following existing educational events whenever possible in order to minimize barriers to participation and reinforce procedural maintenance as a routine component of faculty professional development. Six sessions were conducted following faculty meetings or faculty retreats, while the remaining sessions were conducted following grand rounds. This approach leveraged faculty members' existing educational commitments and minimized the need for additional travel or scheduling accommodations.

Participation was incentivized through continuing medical education (CME) credit and alignment with value‐based incentive (VBI) compensation, reinforcing institutional commitment to ongoing competency. Completion of the HALO training requirement was mandatory to receive any value‐based incentive compensation; faculty who did not complete the HALO requirement were not eligible to receive their VBI payment.

A total of 12 discrete training sessions were conducted over the 1‐year study period, occurring approximately one to two times per month between October and May. Individual sessions lasted approximately 30 to 60 min depending on the number of learners. Sessions were capped at four participants to allow for hands‐on practice and individualized feedback. Faculty registered for sessions in advance and received multiple reminder emails to facilitate scheduling and maximize participation. Training was facilitated by 10 separate faculty trainers, six of whom were not residency leadership or routinely involved in simulation‐based education, supporting the feasibility of disseminating the intervention beyond traditional educational leadership roles. Among the six faculty trainers who were not residency leadership or routinely involved in simulation‐based education, there was representation across junior, mid‐career, and senior faculty (two assistant professors, two associate professors, and two full professors). One faculty trainer facilitated three sessions, while most trainers facilitated a single session. This structure allowed flexibility in participation and broad reach across the department.

All participating faculty completed presession surveys assessing prior experience with transvenous pacemaker placement and baseline comfort. Post‐session surveys assessed changes in comfort, perceived usefulness, and simulator realism. A four‐point Likert scale was intentionally selected to eliminate a neutral response option and encourage participants to indicate whether they felt comfortable or uncomfortable performing the procedure independently. The study was approved under an institutional educational umbrella IRB protocol [20].

## 6 Outcomes

Fifty‐four of 56 eligible faculty members participated in the train‐the‐trainer simulation sessions and completed paired pre‐ and post‐intervention surveys. Baseline data demonstrated limited exposure to TVP placement: 37% reported no prior simulation experience, 24% reported no prior live TVP placement, and 13% reported no experience in either setting.

Faculty‐reported comfort with independently placing a TVP improved substantially following the intervention (see Figure 1). Prior to the session, only 14.8% of faculty reported feeling “comfortable” or “very comfortable.” Post‐intervention, this proportion increased to 83.3%. Mean comfort scores increased from 2.1 ± 0.6 to 3.2 ± 0.6 on a 4‐point Likert scale (*p* < 0.001).

**FIGURE 1:**
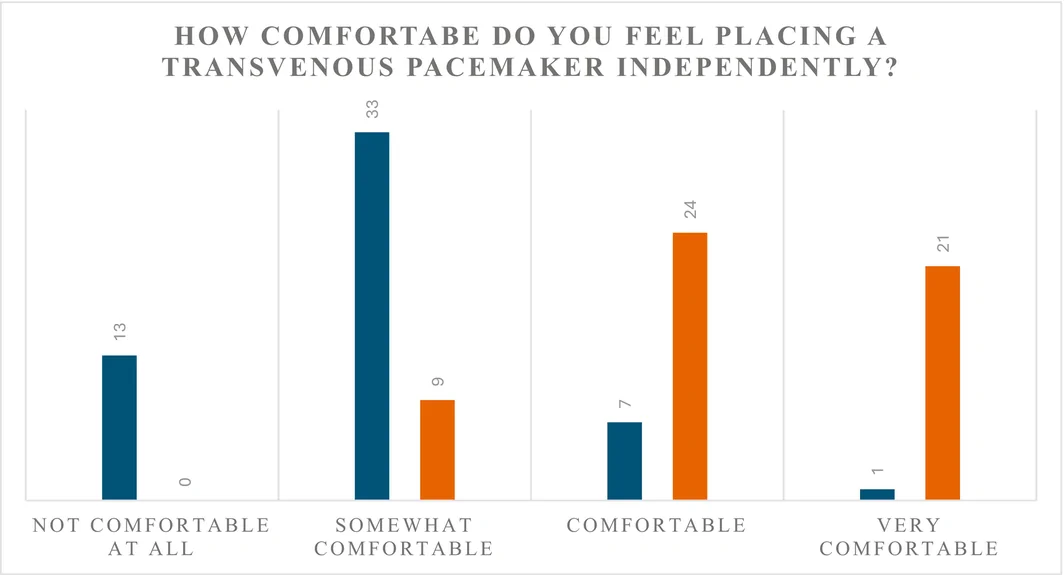
Faculty responses regarding comfortable with independent placement of transvenous pacemaker pre‐intervention (blue) versus post‐intervention (orange).

The training sessions were rated as highly valuable, with a mean usefulness score of 4.9 ± 0.3 on a 5‐point Likert scale. All participants indicated they would recommend the training to colleagues. Among faculty with prior live TVP experience, 61% rated the simulator as “realistic” or “very realistic,” supporting its acceptability as a peer‐teaching tool.

## 7 Reflective Discussion

This innovation demonstrates that a train‐the‐trainer, peer‐led simulation model can effectively support faculty procedural development for HALO procedures. By decentralizing expertise and empowering faculty to teach one another, this approach addresses common barriers to faculty development, including limited time, scarce simulation resources, and sustainability challenges [17].

Several features contributed to the success of this model. Embedding sessions into existing educational structures normalized participation and reduced logistical burden. Peer facilitation fostered a collegial learning environment and aligned with adult learning principles emphasizing relevance and autonomy [18]. Incentivizing participation through CME credit and institutional compensation structures further reinforced the importance of ongoing procedural competency. Importantly, the intervention demonstrated that faculty trainers did not need to be simulation experts or procedural specialists. Instead, standardized trainer preparation and instructional materials enabled faculty across a range of academic ranks and educational backgrounds to successfully facilitate procedural refresher sessions.

While improved self‐reported comfort does not equate to procedural mastery, confidence is a critical prerequisite for timely action in high‐risk clinical scenarios. Faculty discomfort with rarely performed procedures may lead to delays, avoidance, or over‐reliance on consultants. By addressing confidence gaps, this innovation supports procedural readiness and aligns with broader patient safety goals [6, 7]. Implementation also yielded an unanticipated systems‐level benefit by identifying inconsistencies in transvenous pacing equipment across clinical practice sites, allowing these discrepancies to be corrected before clinical use. Although not a predefined outcome, this finding highlights the potential for faculty procedural training initiatives to improve both clinician preparedness and institutional readiness.

This work has limitations. Outcomes were limited to self‐reported comfort, and we did not include objective assessments of procedural performance or long‐term skill retention. Future iterations could incorporate validated mastery checklists, longitudinal refresher intervals, and evaluation of downstream effects on resident education [15, 21]. Additionally, this innovation was implemented at a single institution, which may limit generalizability. Finally, implementation required an initial investment of faculty time to develop standardized trainer materials, assemble the simulation cart, and conduct train‐the‐trainer sessions. Although these activities required approximately 5–10 h of preparatory effort, this investment was incurred once and subsequently supported repeated faculty‐led training sessions with minimal ongoing preparation.

Despite these limitations, the train‐the‐trainer framework described here is highly adaptable and scalable. It offers a practical approach for academic emergency medicine departments seeking to operationalize their responsibility to maintain faculty competency across a range of HALO procedures. By empowering faculty to teach their peers using standardized educational materials and a reproducible training process, this innovation contributes to the growing literature on sustainable models for faculty procedural maintenance. This train‐the‐trainer model provides one practical approach for maintaining faculty competency in HALO procedures by decentralizing procedural expertise and enabling scalable faculty development without reliance on dedicated simulation specialists.

## Author Contributions

**Erin McDonough:** conceptualization, investigation, methodology, project administration. **Sally A. Santen:** conceptualization, investigation, writing – review and editing, supervision. **Elizabeth Leenellett:** conceptualization, investigation, methodology, writing – review and editing. **Jessica Baez:** conceptualization, investigation, writing – original draft, methodology, validation, writing – review and editing, project administration, data curation, supervision, resources. **Heidi Sucharew:** formal analysis.

## Funding

The authors have nothing to report.

## Conflicts of Interest

The authors declare no conflicts of interest.

## Supporting information


**Data S1:** aet270236‐sup‐0001‐supinfo.docx.

## Data Availability

The data that support the findings of this study are available from the corresponding author upon reasonable request.
