# Supplementary material for: Faculty Development for High‐Acuity Procedures: A Train‐the‐Trainer Simulation‐Based Innovation for Transvenous Pacemaker Placement
Source: AEM Educ Train. 2026 Sep 1;10(5):e70236. doi: 10.1002/aet2.70236 (PMC13531364; doi:10.1002/aet2.70236)
Supplement: Supplementary file 1 [file AET2-10-e70236-s001.docx]

**Transvenous Pacemaker Placement**

Set-up Checklist for Trainer

- Grab the cart labeled “transvenous pacer simulation” located in 1654E.
- Top shelf (the “patient”):
  - Fill the gray bucket to marked line with water.
  - Place the motor in marked location. Ensure it is completely submerged in water.
  - Insert the striped end of PVC pipe onto the motor.
  - Plug in motor.
- Middle shelf (the pacing wire):
  - Remove the transvenous pacer from box and arrange in way accessible to proceduralist.
- Bottom shelf (the introducer):
  - Ensure foil tin is filled with water up to level of “skin.”
  - Grab sim lab ultrasound and plug in at simulation location.
  - Place each line (MAC and small introducer) in near foil tin and ultrasound for proceduralist to use if they wish to practice line placement.
- Grab the pacer box located in glass cabinet outside SRU 1. Inform ED team that you have it and provide your contact info in case a patient requires it.
  - Ensure it contains pacer box and connector wires.
  - Place items next to the “patient” for proceduralist to use.
- Optional: You can download a patient monitor smartphone app which allows you to use your device as a patient monitor in order to demonstrate different heart rates and show the proceduralist in real time how a heart rate would change when the pacer is correctly placed. “Simpl” is cheap and user friendly. It helps if you have two devices like a smartphone and tablet.

Clean-up Checklist

- Please ensure pacing wire balloon is deflated prior to removal from sim model.
- Ensure all components are placed back how you found them so that next leader can replicate the steps.
- Dump out water from the gray container and the PVC pipes to prevent leakage. This will require tipping the model upside down and sideways a few times to get all the water out.
- Place supplies back in room and ensure pacer generator goes back to the ED.

**Transvenous Pacemaker Placement**

Steps for Proceduralist

Key: Blue is sterile proceduralist, red is non-sterile proceduralist (the trainer)

- Have learner fill out pre-survey
- Place introducer:
  -
MAC catheter: Located in supply closet. Contains everything needed. Downside is slightly bigger line, so there may be some leakage near where pacing wire enters the introducer.
  - Small introducer: Located in TV pacer box. Would also need to grab a standard central line kit for needle, vascular access kit for sterile field, ultrasound probe cover, and flushes, and a sheath for pacing wire.
  - Either line can be placed on the foil container using ultrasound guidance. Remember that the catheter and dilator are placed simultaneously, contrary to a standard central line placement.
- Check pacing wire:
  -
Test balloon with 1.5cc syringe to ensure it is functional.
  - Practice locking and unlocking balloon by sliding the purple lock. If the arrow aligns, it is unlocked, and the balloon can be inflated or deflated. If arrow does not align, it is locked.
  - Slide sheath over the wire. The side with a small divot is the side that connects to the introducer, so ensure sheath is applied correctly.
- Attach red connectors:
  - These are typically found in the TV pacer box but live with the pacing wire on the sim model.
- Connect pacing wire to pacing generator:
  - Using one of the cords available in the pacing box, connect the cord to the ventricular output on pacing box.
  - Place the proximal lead (labeled on the line) in the positive port of the cord.
  - Secure this in place by twisting the respective side wheel.
  - Place the distal lead in the negative port of the cord.
  - Secure this in place by twisting the respective side wheel.
- Insert pacing wire into the introducer:
  - Advance until 20cm (2 tick marks on the wire).
  - Blow up balloon and lock.
- Turn on pacer generator:
  - It will default to the mode you want.
  - Increase the ventricular output all the way up (25mA) to ensure capture.
  - Can adjust rate as needed. Ensure it is above the rate of transcutaneous pacing to help identify when capture occurs.
- Advance pacing wire:
  - Advance slowly until capture occurs. On this sim model, that occurs when the balloon can be seen in the small tube by the heart.
  - Deflate balloon to ensure no advancement of the pacing wire.
  - Note depth at which capture occurs and include in procedure note.
  - Extend sheath to cover entire pacing wire and secure it at the introducer by connecting divot on sheath to the small dimple on the introducer.
- Minimize electricity:
  - Slowly decrease amount of electricity delivered until capture is lost, then increase by 2mA to ensure continuous capture.
  -

- Have learner fill out post-survey
